# Superiority and clinical significance of Lunx mRNA in the diagnosis of malignant pleural effusion caused by pulmonary carcinoma

**DOI:** 10.1186/1756-9966-32-37

**Published:** 2013-06-08

**Authors:** Ying Tang, Lijun Xu

**Affiliations:** 1 Department of Respiratory Medicine, the First Hospital of Jilin University, Changchun; 130021, China

**Keywords:** Malignant pleural effusion, Pulmonary carcinoma, Lunx mRNA

## Abstract

**Background:**

Pulmonary carcinoma is the main cause of malignant pleural effusions (MPEs). However, there is no satisfactory marker for diagnosing MPEs caused by pulmonary carcinoma. The purpose of this study is to assess the clinical significance of Lunx mRNA detection in diagnosing MPEs caused by pulmonary carcinoma.

**Methods:**

A total of 209 patients with pleural effusions were recruited. The patients were diagnosed by cast-off cells, bronchoscopy, and pleural biopsy. The levels of Lunx mRNA in the pleural effusions were determined by real-time PCR. The levels of PH, LDH, glucose, albumin, and CEA were also determined. Patients who accepted chemotherapy underwent Lunx mRNA detection before and after the first chemotherapy session. The patients were divided into four groups according the effect of chemotherapy: complete remission (CR), partial remission (PR), no change (NC), and progressive disease (PD). The patients were also divided into two groups according the change in direction of Lunx mRNA expression after chemotherapy: increased group and decreased group. The patients were followed up to determine survival.

**Results:**

Lunx mRNA was positive in 89 of 106 patients with pleural effusions caused by pulmonary carcinoma. The specificity and sensitivity were 95.9% and 84.9%. The area under the ROC curve was 0.922. Lunx mRNA detection was better than detection using cast-off cells and CEA. All of the Lunx-positive patients with MPEs were diagnosed with pulmonary carcinoma, and all extrapulmonary carcinoma patients were Lunx-negative. The positive predictive value of Lunx mRNA for the source of tumor cells was 100%. Lunx mRNA expression decreased after the first session of chemotherapy in the CR and PR groups, increased in the PD group, there was no change in the NC group. Further analysis indicated the change in direction of Lunx mRNA expression was associated with the overall survival of patients. The patients in the increased group had longer overall survival times than those in the decreased group.

**Conclusion:**

Lunx mRNA is a specific tumor gene that is highly expressed in MPEs caused by pulmonary carcinoma. The changes in Lunx mRNA levels after chemotherapy can predict the prognosis of patients with MPEs caused by pulmonary carcinoma.

## Background

Pleural effusion is a common disease that is caused by pulmonary carcinomas and other malignant tumors, such as breast cancer and ovarian cancer, and even some nonmalignant diseases including tuberculosis and pneumonia [1,2]. Malignant pleural effusion (MPE) is usually associated with cancer-related mortality and morbidity. Thus, it is important to diagnose MPEs and to treat and evaluate prognosis.

Cytology detection is the conventional method used to distinguish tumor cells in pleural effusions, as described in the International Union Against Cancer/American Joint Committee on Cancer’s tumor-node metastasis (TNM) classification system [3]. However, cytology detection is imperfect in diagnosing MPEs. Moreover, when pleural effusion cytology cannot establish a patient’s diagnosis, additional invasive procedures must be performed to sample pleura for histological examination to enhance the diagnostic rate [2]. However, there are high risks associated with these procedures, and many hospitals do not have these technologies, which limits their clinical application. Therefore, the diagnosis of MPE presents challenges to clinicians, and it is urgent to search for an effective diagnostic biomarker for this disease.

Lung cancer markers, including carcinoembryonic antigen (CEA), neuron-specific enolase (NSE), squamous cell carcinoma (SCC) antigen, and cytokeratin 19 (CK19), have been generally utilized to identify malignant and nonmalignant pleural effusions [4-7]. However, the diagnostic utility of these markers is unsatisfactory. Lung-specific X protein (Lunx), which was isolated by Yoshiyuki and colleagues through differential-display mRNA analysis, is a 206 bp cDNA fragment specifically amplified in the lung [8]. The Lunx gene consists of 1,015 nucleotides, including an open reading frame of 768 nucleotides that encodes 256 amino acids [8]. Lunx has served as a useful molecular marker for the detection of pulmonary carcinoma in bronchial brushing specimens and for the detection of peripheral blood and lymph node metastases [9-12]. Cheng et al. [13] reported that Lunx mRNA was the most specific biomarker with the highest sensitivity when compared with CK19, CEA, vascular endothelial growth factor-C (VEGF-C), and heterogeneous ribonuclear protein (hnRNP) for the differential diagnosis of non-small cell lung cancer from pleural effusion. However, it is still unclear whether Lunx mRNA expression in pleural effusions can predict the source of tumor cells and the responses of patients to chemotherapy.

Reverse transcriptase polymerase chain reaction (RT-PCR) is the most sensitive method for the detection of micrometastatic diseases, allowing for the detection of one cancer cell in 10^6^ to 10^7^ mononuclear cells [14,15], but it is not effective in evaluating therapeutic effect and prognosis. Quantitative real-time RT-PCR can be used to assess gene expression levels and further evaluate the relationship between genes and disease. Currently, very little information is available on the relationship between the expression of Lunx mRNA and MPE.

The main purpose of this study was to evaluate Lunx mRNA expression in lung cancer cells using quantitative real-time RT-PCR, and to assess the diagnostic usefulness of Lunx mRNA expression as a tumor marker in pleural effusion. Furthermore, the correlation of Lunx mRNA expression in pulmonary carcinoma patients with pleural effusion and clinical factors was investigated.

## Methods

### Patients and controls

Two hundred and nine patients with pleural effusions were recruited from the inpatient hospital of the First Hospital of Jilin University from July 2010 to January 2013. MPEs were diagnosed in 112 patients. Of these patients, 106 cases were pathologically shown to have pulmonary carcinoma and six patients had extrapulmonary carcinoma. Four patients with pathologically proven pulmonary carcinoma of the lung did not have MPEs. The pleural effusions of three of these patients were caused by heart failure, and the other was caused by hypoproteinemia. The other 93 patients were diagnosed with nonmalignant pleural effusions, including 42 caused by tuberculosis, 13 caused by pneumonia, and 38 caused by heart failure or hypoproteinemia. The clinical characteristics of the patients are shown in Table 1. Eighty-two patients accepted chemotherapy (Table 2), and the therapeutic effect was evaluated after two sessions of treatment. The 82 patients received first-line chemotherapy regimens for non-small cell lung carcinoma (NSCLC), including navelbine plus cis-platinum or carboplatin (NP), paclitaxel plus cis-platinum or carboplatin (TP), gemcitabine plus cis-platinum or carboplatin (GP), or docetaxel plus cis-platinum or carboplatin (DP), or they received a chemotherapy regimen for small cell lung carcinoma (SCLC), namely etoposide plus cis-platinum (EP). Lunx mRNA expression was detected before and after the first session of chemotherapy. The study was approved by the Human Ethics Committee of Jilin University, Changchun, China. Written informed consent was obtained from each participant.

**Table 1:** Clinical characteristics of patients

**Patients with pleural effusion**	**Pulmonary carcinoma (n = 110)**		**Pneumonia (n = 13)**	**Tuberculosis (n = 42)**	**Heart failure/hypoproteinemia(n = 38)**	**Extrapulmonary carcinoma (n = 6)**
	**Malignant (n = 106)**	**Nonmalignant (n = 4)**				
Age ($\bar{x}$ ± S)	63.35 ± 9.35	61.25 ± 6.29	46.23 ± 11.56	43.38 ± 13.88	64.78 ± 8.53	51.17 ± 9.13
Sex (M/F)	55/51	2/2	7/6	20/22	20/18	2/4
Cast-off (N/P)	38/68	4/0	13/0	42/0	38/0	3/3
Pleural biopsy (n)	49	4	—	8	—	3
TNM stage						
I	—	3	—	—	—	1
II	—	1	—	—	—	1
III	—	—	—	—	—	—
IV	106	—	—		—	4
Pathological type						
	SCC 26	—	—	—	—	Hepatoma 2
	Ade 71	—	—	—	—	ovarian cancer 1
	SCLC 9	—	—	—	—	pleural endotheliomas 1
			—	—	—	breast cancer 2
Pleural effusion ($\bar{x}$ ± S)						
PH	7.42 ± 0.05	7.45 ± 0.02	7.18 ± 0.04	7.36 ± 0.04	7.45 ± 0.05	7.48 ± 0.03
LDH	665.48 ± 226.18	203.25 ± 57.64	363.46 ± 64.7	384.93 ± 93.44	135.79 ± 32.38	575.5 ± 152.28
Glu	4.52 ± 0.81	4.87 ± 0.3	4.78 ± 0.53	4.7 ± 0.58	4.74 ± 0.36	4.46 ± 0.77
Alb	46.59 ± 4.84	24.11 ± 1.57	42.47 ± 5.05	47.57 ± 4.59	22.15 ± 2.28	47.93 ± 4.63

Extrapulmonary carcinoma: including breast cancer, pleural endotheliomas, and lymphadenoma; *N/P* negative/positive, *SCC* squamous cell carcinoma, *Ade* adenocarcinoma, *SCLC* small cell lung cancer, *PH* power of hydrogen, *LDH* lactate dehydrogenase, *Glu* glucose, *Alb* albumin —: no data.

**Table 2:** Clinical characteristics and therapeutic effects in patients with MPE caused by pulmonary carcinoma

**Group**	**CR**	**PR**	**NC**	**PD**
Case number	12	48	10	12
Age ($\bar{x}$ ± S)	61.16 ± 8.87	63.5 ± 9.85	63.7 ± 6.36	66.92 ± 10.92
Sex (M/F)	8/4	23/25	6/4	3/9
Pathological type				
SCC	3	10	8	3
Ade	8	35	1	9
SCLC	1	3	1	0

*CR* complete remission, *PR* partial remission, *NC* no change, *PD* progressive disease, *SCC* squamous cell carcinoma, *Ade* adenocarcinoma, *SCLC* small cell lung cancer.

### Bronchoscopy

Patients with pleural effusions who showed a lump in pulmonary computed tomography (CT) underwent bronchoscope detection. They received topical anesthesia with 5 ml of 2% lidocaine inhaled for 10–15 minutes and 2 ml of 2% lidocaine dropped in each nostril. The bronchoscope was inserted nasally with the patients in the supine position. During the procedure, endobronchial or transbronchial biopsy specimens were collected for histopathology. Their specimens were sent to the department of pathology for pathology detection by a trained specialist.

### Detection of cast-off cells from pleural effusions

All patients underwent thoracentesis during hospitalization, and 300–500 ml of pleural effusion was inspired from the indicated patients. Then the effusion was centrifuged at 3000 rpm for 8 min to pellet cells. The supernatant of the effusion was removed, and the pellet of pleural effusion cells was resuspended. Each sample was smeared onto 6–8 glass slides, and fixed. Following hematoxylin-eosin staining, the cell types were observed using a microscope. The above steps were also completed by a trained specialist.

### Pleural biopsy

Patients who did not undergo bronchoscopy or who had positive endobronchial or transbronchial biopsy results and repeatedly tested negative for cast-off cells in the pleural effusion underwent pleural biopsy. The puncture site was chosen by ultrasound. After routine disinfection and draping, 2% lidocaine was subcutaneously injected for local anesthesia. Then the pleural biopsy needle was inserted into the pleural cavity via a 0.5 cm epidermal incision. When the needle was definitely established in pleural cavity, a hooked, blunt acupuncture needle was inserted into the chest along the needle guard, and 3–4 left, right, and subtus parietal pleura tissues were aspirated. The tissues were fixed with dilute formaldehyde for further pathological examination.

### Clinical parameters of pleural effusion

Five milliliters of pleural effusion were inspired from each of the patients. The power of hydrogen (PH) was determined with a blood gas machine (ABL700, Radiometer Medical A/S, Denmark). The levels of lactate dehydrogenase (LDH), albumin (Alb), and glucose (Glu) were determined with a biochemistry analyzer (AU400, Olympus, Japan). The CEA values were determined by the chemiluminescence immunoassay method (Beckman Coulter, Inc., Fullerton, United States) with the upper limit of 5 ng/ml in normal adult.

### Lunx detection via real-time PCR

The pleural effusion sample (15 ml) was centrifuged at 3500 rpm for 10 min to pellet cells. Then the total cellular RNA was extracted using the Trizol reagent according to the protocol provided by the manufacturer. Lunx detection was performed using a Lunx mRNA fluorescence PCR diagnostic kit (China, Anhui Puyuan Biology Technology Corporation) according to the protocol provided by the manufacturer. Quantitative real-time PCR was performed using an ABI PRISM 7000 sequence detector (Applied Biosystems, Foster City, United States). The standard RT reaction contained 3.5 μl reverse transcription reaction solution, 5 μl RNA solution, and 1.5 μl water without RNA enzyme in a total volume of 10 μl. The standard PCR contained 5 μl reverse transcription reaction solution, 5 μl RNA solution, and 1.5 μl water without RNA enzyme in a total volume of 25 μl. The initial PCR step was at 50°C for 2 min, followed by a 5 min hold at 95°C. The PCRs were performed using a total of 60 cycles consisting of a 15 s melt at 95°C, followed by a 1 min annealing/extension at 56°C. Each sample was analyzed in triplicate for the target gene and mRNA. Copy numbers less than 10^3^ were considered negative.

### Statistical analysis

SPSS 18.0 software was used to analyze the results of real-time PCR. The K independent samples test was used to compare the gene expression levels in pleural effusion among different groups, to compare pulmonary carcinoma patients in different pathologic groups, and to compare patients before and after clinical treatment. The receiver operating characteristic (ROC) curve was used to compare the specificity and sensitivity among different indexes. In cases where the results of gene expression were negligible, the data were treated as 0 for statistical convenience. The Kaplan-Meier curve was used to analyze the overall survival of patients. A value of *P* < 0.05 (two-tailed test) was considered significant.

## Results

### General gene expression in each group

**Figure 1:**
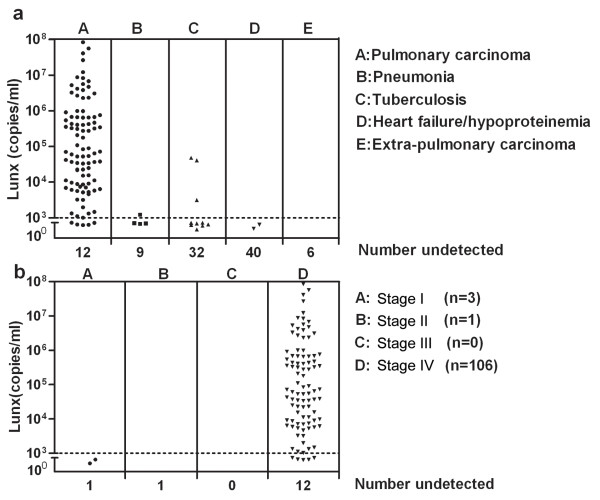
**Lunx mRNA expression in the pleural effusion of indicated patients. a**: Levels of Lunx mRNA in patients with pleural effusions caused by different diseases. **b**: Levels of Lunx mRNA in patients with pleural effusions caused pulmonary carcinoma at different stages. The horizontal line indicates 10^3^ copies/ml of Lunx mRNA. Copy numbers less than 10^3^ copies/ml were considered negative. When the copy number of Lunx mRNA was not detectable, the results were shown as number undetected.

In the present study, we detected the expression of Lunx mRNA in different pleural effusion patients. Lunx mRNA was positively detected in 89 of the 106 patients with pleural effusion caused by pulmonary carcinoma. Lunx mRNA expression was not detected in patients with heart failure/hypoproteinemia or extrapulmonary carcinoma. However, one patient with pneumonia and three patients with tuberculosis were positive for Lunx mRNA expression. The Lunx mRNA expression in different groups is shown in Table 3. The pulmonary carcinoma patients with pleural effusion were grouped by the TNM classification, and there were three patients in stage I, one patient in stage II, and 106 patients in stage IV. The expression levels in different groups are shown in Figure 1.

**Table 3:** Expression of each marker in patients with pleural effusion caused by different diseases

**Group**	**n**	**Lunx**		**Cast-off**		**CEA**	
		**Positive**	**Negative**	**Positive**	**Negative**	**Positive**	**Negative**
Pulmonary carcinoma	106	89	17	68	38	73	33
Pneumonia	13	1	12	0	13	0	13
Tuberculosis	42	3	39	0	42	6	36
Heart failure/hypoproteinemia	42	0	42	0	42	3	39
Extrapulmonary carcinoma	6	0	6	3	3	5	1

### RT-PCR detection of Lunx mRNA was superior to the detection of cast-off cells and CEA in diagnosing MPEs caused by pulmonary carcinoma

**Figure 2:**
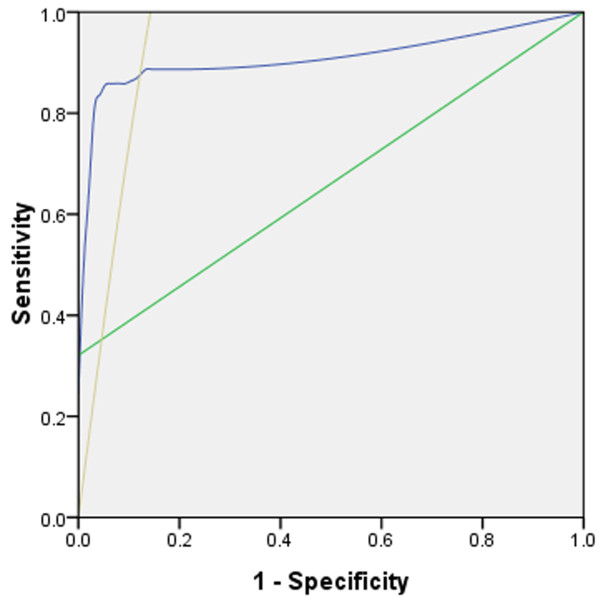
**ROC curve of Lunx mRNA, cast-off cells, and CEA.** The specificities for detecting Lunx mRNA, cast-off cells, and CEA are 95.9%, 100%, and 90.7%, respectively. The sensitivities for detecting Lunx mRNA, cast-off cells, and CEA are 84.9%, 64.2%, and 68.9%, respectively. The area under the ROC curves for detecting Lunx mRNA, cast-off cells, and CEA are 0.922, 0.821, and 0.798, respectively. Blue: Lunx mRNA; Green: cast-off cells; Brown: CEA.

The detection of cast-off cells and CEA are commonly used methods for diagnosing MPEs. Therefore, we compared the efficiency of Lunx mRNA, cast-off cells, and CEA detection in diagnosing MPEs caused by pulmonary carcinoma and nonmalignant pleural effusions. Lunx mRNA was positively detected in 93 of 209 patients with pleural effusions. Of these patients, four were diagnosed with nonmalignant pleural effusions, and the others were diagnosed with MPEs caused by pulmonary carcinoma (Table 3). CEA was positively detected in 87 of 209 patients with pleural effusions. Of these patients, 73 were diagnosed with MPEs caused by pulmonary carcinoma, and nine patients were diagnosed with nonmalignant pleural effusions (Table 3). Sixty-eight patients with pleural effusions caused by pulmonary carcinoma were positive for cast-off cells in the pleural effusions. The specificities of detecting Lunx mRNA, cast-off cells, and CEA were 95.9%, 100%, and 90.7%, respectively. The sensitivities of detecting Lunx mRNA, cast-off cells, and CEA were 84.9%, 64.2%, and 68.9%, respectively. The area under the ROC curve for Lunx mRNA, cast-off cells, and CEA detection were 0.922, 0.821, and 0.798 (Figure 2). The optimal threshold for Lunx mRNA detection according to the ROC analysis was 985 copies, and it was similar to our positive threshold.

### The relationship between the levels of Lunx mRNA and the degree of tumor cell differentiation in pulmonary carcinoma

**Figure 3:**
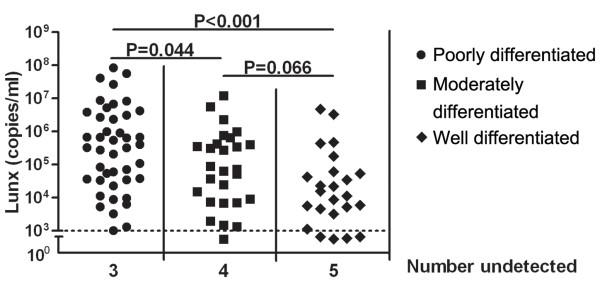
**Lunx mRNA expression according to tumor differentiation.** Lunx mRNA was detected by real-time RT-PCR. Levels of Lunx mRNA in poorly, moderately, and well differentiated groups. The horizontal line indicates 10^3^ copies/ml of Lunx mRNA. Copy numbers less than 10^3^ copies/ml were considered negative. When the copy number of Lunx mRNA was not detectable, the results were shown as number undetected.

According to the National Comprehensive Cancer Network (NCCN) Clinical Practice Guidelines in Oncology [16], most pleural effusions associated with lung cancer should appear in stage IV. The effusion is not related to the tumor in only a few patients who have had multiple cytopathologic pleural effusion examinations that are negative for tumor cells, and when the effusion is nonbloody and not an exudate. Pleural effusion unrelated to the tumor should be excluded as a stage element. In this study, the numbers of cases in stage I, II, and III were small, so the statistical power was insufficient when comparing the relationship between gene expression and TNM stage. Furthermore, we examined the relationships between the levels of Lunx mRNA and PH, LDH, glucose, albumin in the pleural effusion, histopathological category, and the degree of tumor cell differentiation, which referred to the degree of tumor cell differentiation close to normal cells. There was no association between the levels of Lunx mRNA and PH, LDH, glucose, and albumin in the pleural effusion (data not shown). Also, no difference was found in Lunx expression in the different histopathological categories (data not shown). The levels of Lunx mRNA expression were higher in poorly differentiated than in moderately differentiated and well differentiated tumors (*P* = 0.044, *P* < 0.001, respectively, Figure 3). There was no statistical difference in Lunx mRNA expression between moderately and well differentiated tumors (*P* = 0.066, Figure 3).

### Lunx mRNA expression can predict the source of tumor cells

Lunx mRNA expression is specific to the lungs, but it was unknown whether it could predict the source of tumor cells in MPEs caused by pulmonary carcinoma. We analyzed Lunx mRNA expression in patients with MPEs. There were 112 patients diagnosed with MPE including 106 pulmonary carcinoma and 6 extrapulmonary carcinoma patients. All of the Lunx-positive patients were diagnosed with pulmonary carcinoma, and all of the Lunx-negative patients were diagnosed with extrapulmonary carcinoma (Table 3). The positive predictive value for Lunx was 100%.

### Changes in Lunx mRNA expression were associated with the response of patients to chemotherapy

**Figure 4:**
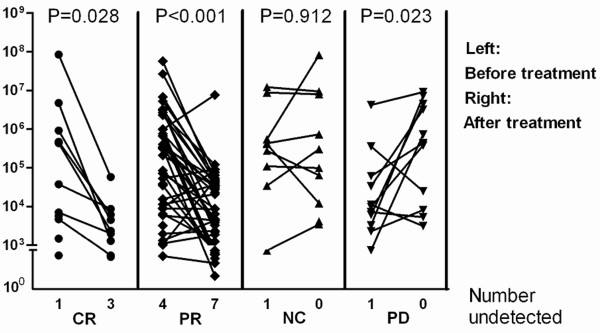
**Lunx mRNA expression in the pleural fluid before and after the first chemotherapy session.** Pleural fluid samples from 82 patients were collected before and after treatment and divided into the CR, PR, NC, and PD groups. Copy numbers less than 10^3^ copies/ml were considered negative. When the copy number of Lunx mRNA was not detectable, the results were shown as number undetected. CR: complete remission, n = 12; PR: partial remission, n = 48; NC: no change, n = 10; PD: progressive disease, n = 12.

The 82 patients who accepted chemotherapy underwent Lunx detection before and after the first chemotherapy session. The relationship between the change in Lunx mRNA expression and the response to chemotherapy was evaluated. The standard therapeutic effect was measured according to the WHO criterion [17]. Following chemotherapy, 12 patients had complete remission (CR), 48 patients had partial remission (PR), 10 patients had no change (NC), and 12 patients had progressive disease (PD). The Lunx expression decreased after the first session of chemotherapy in the CR and PR groups (*P* = 0.028, *P* < 0.001, respectively), there was no change in the NC group (*P* = 0.912), and there was an increase in the PD group (*P* = 0.023) (Figure 4).

### Changes in direction of Lunx mRNA expression were associated with the overall survival of patients

**Figure 5:**
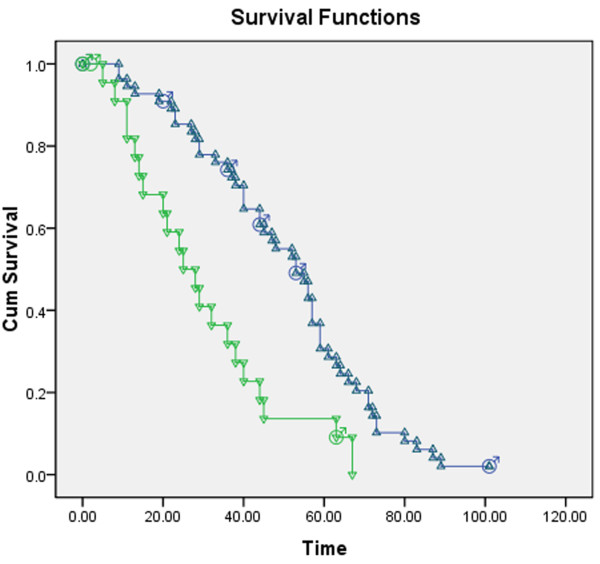
**Overall survival curves of patients after chemotherapy.** Patients were divided into the increased Lunx mRNA expression group and decreased Lunx mRNA expression group according the direction of change in Lunx mRNA expression. One patient was lost to follow-up and five patients were alive in the increased Lunx mRNA expression group, and two patients were lost and one patient was alive in the decreased Lunx mRNA expression group. Time was calculated in weeks. The overall survival curves are shown in blue for the increased Lunx mRNA expression group and in green for the decreased Lunx mRNA expression group. The individual participants are represented as triangles. The censored data are represented by the male symbol.

Overall survival is the best index to confirm the effectiveness of therapy. Change in Lunx mRNA expression were associated with the responses of patients to chemotherapy. Therefore, it was important to assess whether the change in Lunx mRNA expression was associated with the overall survival of patients. The patients who accepted chemotherapy were divided into two groups according the direction of change in Lunx mRNA expression: increased Lunx mRNA expression group and decreased Lunx mRNA expression group (Figure 5). Two patients with negative Lunx expression both before and after treatment were excluded from the analysis. There were 6 censored data (1 lost and 5 survival) in the increased Lunx mRNA expression group, and 3 censored data (2 lost and 1 survival) in the decreased Lunx mRNA expression group. The median overall survival was 53 weeks (95% confidence interval [CI] 44.003–61.997) in the increased Lunx mRNA expression group, and it was 25 weeks (95% CI 15.807–34.193) in the decreased Lunx mRNA expression group. The patients in the increased Lunx mRNA expression group had longer overall survival times than those in the decreased Lunx mRNA expression group (*P* = 0.000).

## Discussion

The production of MPE is a pathological process, which results from the failure of pleural defense mechanisms and abnormal mesothelial function, and it is defined by the presence of tumor cells in the pleural effusion [18]. Pulmonary carcinoma is one of the main causes of MPE [19,20]. Patients with pleural effusion caused by pulmonary carcinoma often have a short median survival [21]. The etiological diagnosis of pleural effusions is important for evaluating the prognosis of patients. However, the current diagnostic tests for MPEs are still unsatisfactory.

Lunx mRNA is expressed in normal lung tissues and pulmonary carcinoma tissues, but not in other normal or tumor tissues [8], and it has served as a useful molecular marker for the detection of pulmonary carcinoma [11,13,22]. However, little information is available on the role of Lunx mRNA expression in the diagnosis of pleural effusions caused by pulmonary carcinoma. In the present study, we found that Lunx mRNA expression was positively detected in 89 of 106 patients with pleural effusions caused by pulmonary carcinoma, and the area under the ROC curve for Lunx mRNA detection was 0.922. The diagnostic utility of Lunx mRNA expression is superior to the use of cast-off cells and CEA. These data provide firm evidence that the detection of Lunx mRNA expression in pleural effusion via RT-PCR is a specific and sensitive method for diagnosing MPEs caused by pulmonary carcinoma, and our results agree with those of Cheng et al. [13].

Hyperplastic mesothelial cells, rhagiocrine cells, and degenerative mesothelial cells often display special morphological characteristics in the pleural effusion, which makes it difficult to identify the source of the tumor cells [23]. In addition, tumor cells partially lose their characteristics when they unrestrictedly passage in the pleural effusion [24]. Therefore, it is important to find markers to distinguish the source of tumor cells. Lunx mRNA is specifically expressed in the lungs, so we hypothesized that positive Lunx mRNA expression may serve as a tool to identify the source of tumor cells in pleural effusions. In the present study, all Lunx-positive patients with MPEs were diagnosed with pulmonary carcinoma, and all extrapulmonary carcinoma patients were Lunx-negative. From the above results, we conclude that if the Lunx mRNA expression in a patient with MPE is positive, then the source of the tumor cells should be the lungs.

Lunx mRNA is an effective marker of pulmonary carcinoma. In the present study, we analyzed the relationship between Lunx mRNA expression and clinical parameters. We found no association between the levels of Lunx mRNA expression and LDH levels, glucose levels, albumin in the pleural effusion, PH, or histopathological category. However, there were significantly increased levels of Lunx mRNA expression in poorly differentiated tumors compared to moderately and well differentiated tumors. The degree of tumor cell differentiation is recognized as one index to evaluate prognosis. We presume that Lunx mRNA expression levels may be associated with the prognosis of patients with MPE caused by pulmonary carcinoma.

Once diagnosed, chemotherapy is the main method to treat patients with MPE caused by pulmonary carcinoma [16]. In the CR and PR groups, we found that the expression of Lunx mRNA was significantly decreased after the first session of chemotherapy. There was no significant difference in the NC group; however, the expression of Lunx mRNA significantly increased in the PD group. These data indicate that the change in Lunx mRNA expression may be associated with the patients’ response to chemotherapy and that Lunx mRNA expression is an effective index for evaluating the effect of chemotherapy. To investigate this idea further, we divided the patients who accepted chemotherapy into two groups according the change in direction of Lunx mRNA expression, and investigated the overall survival of the patients. We found that the patients in the increased Lunx mRNA expression group had longer overall survival times than those in the decreased Lunx mRNA expression group. These data indicated that the change in direction of Lunx mRNA expression after chemotherapy can predict the prognosis of patients.

## Conclusions

In conclusion, Lunx mRNA is a specific tumor gene that is highly expressed in MPE caused by pulmonary carcinoma. The detection of Lunx mRNA before and after chemotherapy can help clinicians predict the prognosis of patients. Lunx mRNA is a sensitive marker for distinguishing MPEs caused by pulmonary carcinoma from pleural effusions caused by other reasons. This detection may lead to the early diagnosis of patients with MPE caused by pulmonary carcinoma.

## Competing interests

The authors declare that they have no competing interests.

## Authors’ contributions

Y T carried out the experiments and drafted the manuscript. LJ X designed the experiments. Both authors read and approved the final manuscript.
